# Experiments, Relative to the Chemical Analysis of Opium

**Published:** 1803-05-01

**Authors:** 

**Affiliations:** Apothecary, at Erfurt


### EXPERIMENTS, *relative to the* CHEMICAL ANALYSIS *of* OPIUM; *by* MR. BUCHHOLZ, *Apothecary, at Erfurt.*

THE accurate chemical knowledge of so curious a substance as opium is a matter of great importance, particularly as it may throw some light on its mode of operating in the animal body, or inform us at least, which of its constituent particles is the most active, and thus direct our attention towards a method of obtaining the most efficacious preparation of so powerful and useful a medicine. The chemical analysis of this substance, however, though it has been undertaken by many chemists, deserves, on the whole, to be deemed imperfect; a circumstance for which we may easily account, on considering how much the chemical treatment of vegetable substances was formerly limited, being by no means calculated to explore the composition and number of their constituents, and to afford atisfactory results. Induced by these considerations, and persuaded of the deficiency of all the former analyses of opium, I determined to attempt a new one, which, however distant from being entirely satisfactory, will, I trust, prove the first step towards a more exact knowledge of the chemical mical mixture of so remarkable a substance, than we have hitherto possessed.

*Previous analysis of opium.* I extracted two ounces of the very best opium, according to the prescription of the Edinburgh Dispensary, by means of equal parts of proof spirit and cinnamom water. The residuum remaining after this operation, amounted to three drachms and a half, which I treated repeatedly for four times, one after another, with alcohol, as long as this received any colour from it; for which purpose I used 12 ounces of alcohol, and I boiled the residuum with a sufficient quantity of distilled water, which however was not coloured by it. The residuum, after having been thus treated with aclohol and water and well dried, still weighed two drachms 10 grains. On throwing a small portion of this substance on red hot iron it appeared like a black oil, and being cooled, it could be drawn into threads, emitting the smell of horn. Another portion, treated with concentrated nitric acid, gave to it a dark red colour, whereby red fumes, attended with a strong frothing, were disengaged, as soon as it was gently heated. Digested for some hours with rectified vitriolic either, the tincture received a dark brown colour. This solution being mixed with water and shaken, a greasy tough mass was separated, which could be drawn out in long threads, and showed several characteristics of Koutshoock matter. By this preliminary discovery I was encouraged to pursue the path which I had entered, and thus to reach the point which I purposed.

*Accurate analysis of opium.* Exp. I. Five hundred grains of very pure and good opium were cut into small pieces, and being thrown into a retort, were infused with 32 ounces of distilled water. After having been digested for several hours in a heat approaching to the boiling degree, the fire was gradually augmented, whereby 6 ounces of liquor were distilled over, which had a light colour, and a strong peculiar smell of opium, or of the fresh heads of poppy, and a peculiar taste, somewhat similar to that of opium, but less acrid and warm. I could not find the least trace of oil, and having put to the retort a new receiver, I distilled over 6 ounces more of a liquor which had neither smell nor taste.

Exp. II. In order to ascertain whether the smell and taste of the water distilled from opium originated in oily particles, I tried to concentrate them by a gentle distillation; for which purpose I drew two ounces of liquor by a very gentle heat, during which process the peculiar smell of opium was perceived through the laboratory, though the vessels were very exactly luted. This liquor tasted and

 smelled smelled like opium, but no trace of any oily particles could be found out, and it had no effect on the tincture of tournesol. As it has been supposed by some authors, that the principal virtue of opium might be impaired in some measure by boiling and by the evaporation of the volatile particles, I thought to have obtained them in the above-mentioned manner, and accordingly expected a great operation of the distilled liquor on the animal body, on which account I made the following experiment.

Exp. III. Half an ounce of that liquor was administered to a weak sickly dog of two years, but though I watched him closely for more than one hour, I could not perceive any sensible effect from this dose, and having given him the rest of the liquor, about one ounce and a half, no bad effect was produced.

Exp. IV. I separated the extract obtained in the first experiment by water through a filtrum of fine unglued paper, and having well washed the residuum, I let it boil for three hours with 16 ounces of distilled water, which however became but slightly coloured, and had a weak taste of opium. After I had filtrated it, and washed sufficiently with distilled water, I poured it to the liquor obtained in the first experiment.

Exp. V. All these liquors were evaporated in a good earthen vessel by a very gentle heat to about one ounce and a half, which I suffered to stand, well covered, in a moderately warm place for eight days, and the liquor being poured off I found 2 grains of a reddish grey matter, which being somewhat resinous, contained a small portion of lime and of a vegetable acid, for it crackled under the teeth, and being calcined, the residuum was entirely dissolved in nitric acid with a considerable effervescence. The liquor above mentioned was now evaporated by gentle heat to the consistency of opium, and the extract thus obtained weighed 330 grains, or 5 drachms and a half. It was easily pulverised, of a glossy brown surface, and very brittle; its taste was moderately acrid.

Exp. VI. The residuum extracted from the watery solution being well dried, weighed exactly 135 grains, was very brittle, of no considerable taste.

Exp. VII. These 135 grains being infused with 6 ounces of alcohol in a proper matrass, which being shut with a bladder was exposed to digestion. This being continued for two days and the vessel frequently shaken, the mixture obtained a dark brown colour. I filtrated the liquor when still still hot, pouring to it alcohol as long as it run coloured throug the filtrum. The filtrated tincture on being cooled deposited some flakes which I found to consist of a matter like gluten.

Exp. VIII. I poured again 6 ounces of alcohol on the residuum, which I had carefully collected, and having treated the mixture for about two hours in the boiling heat, I let it stand for twelve hours in a moderate warmth. The tincture was likewise strongly saturated, and let fall some flakes, as I have remarked in the foregoing experiment.

Exp. IX. The residuum thus twice extracted with alcohol was a third time infused with 4 ounces of alcohol, and put in digestion for several hours; the tincture became again pretty well saturated.

Exp. X. The residuum was again boiled with 4 ounces of alcohol, and though it was still considerably coloured, it became not in the least turbid by the admixture of water; whence I concluded the colour might rather be attributed to a colouring matter than to any resinous part; which I was the more inclined to believe, as it seemed extraordinary that 20 ounces of alcohol should not have been able to dissolve 2 drachms and 15 grains of a resinous matter. The residuum, along with the precipitated flakes, weighed 90 grains.

Exp. XI. I infused these 90 grains with 1 ounce of vitriolic ether, exposed the mixture to a gentle heat, often shaking it. After being thus digested for about twelve hours, the mixture received a dark brown colour. I filtrated the solution, pouring on the residuum vitriolic ether till it run through uncoloured.

Exp. XII. The residuum was again mixed with one ounce of vitriolic ether, and placed in a moderate warmth, and some time after heated; the tincture however was scarcely coloured. The liquor being separated by the filtrum, was mixed with that obtained in the former experiment. The residuum, which was grey, red, and powdery, weighed dry 66 grains.

Exp. XIII. A small portion of the residuum I threw into a small red hot crucible, by which a disagreable smell, resembling that of burned horn, was disengaged, some coal remaining, which could only with difficulty be calcined.

Exp. XIV. In order to separate the substance, dissolved in the vitriolic ether, I mixed this in a large vessel with with as much distilled water as was sufficient for the solution of the ether, by which means that substance coagulated and collected itself, swimming, in form of a blackish juice on the surface of the liquor. Its quantity amounted to about 24 grains, and it possessed all the characteristics of the Koutshoock matter; thrown on living coals it emitted a disagreeable smell, resembling that of lard. The insoluble residuum, remaining after the treatment with water, alcohol, and vitriolic ether, proved to be nothing but albuminous matter, mixed with fibrous and ligneous particles, which only resists the solution in alcohol, water, and vitriolic ether, and emits the smell of horn.

Exp. XV. One hundred grains of the dry extract, obtained by the evaporation of the watery digestion, being pulverised, were infused with 1 ounce of alcohol, and heated for half an hour, by which the liquor became dark brown. The experiment being repeated with the same quantity of alcohol, produced a strongly coloured tincture; but on its third repetition, I could not obtain a similar tincture, the alcohol remaining uncoloured, and showing no taste of opium.

Exp. XVI. All spirituous solutions were evaporated to dryness by a gentle heat; the mass thus obtained had the appearance of the extract of saffron, but being dried, it became glossy, yellow brown, and brittle, like resin; it was entirely soluble in water, weighed about 54 grains, and was saponaceous; whence it appears, that the 330 gr. of the watery extract contained 178 gr. saponaceous matter and 152 gr. gum.

Exp. XVII. The mass of the watery extract, remaining after its extraction with alcohol, was again dissolved in distilled water and mixed with three or four times as much alcohol, by which means the gum was separated in form of brownish flakes, and the remaining liquor was but slightly tinged, which proves that the gum was quite pure from the saponaceous matter.

Exp. XVIII. A solution of the watery extract of opium was mixed with vitriol of iron, but the brown solution was hardly tinged by it.

Exp. XIX. A similar solution mixed with a solution of muriat of barytes became turbid, and a precipitation ensued. Mixed with sulphat of silver it got slightly turbid; but on adding to it alcohol it remained clear.

Exp. XX. A solution of opium in alcohol became darker when mixed with a solution of vitriol of iron, but not black.

Exp.

Exp. XXI. The remainder of the watery extract from experiment vi. being about 230 grains, were dissolved in distilled water, filtrated and evaporated to the consistency of a syrup, which being left to dry in a very moderate warmth for about three weeks yielded a dry mass, on which not any crystalline particles could be perceived.

Exp. XXII. I mixed together the extracts made with alcohol in the experiments viii. ix. x. and xi. put them into a small retort and distilled over the alcohol to its fourth part, during which operation a small portion of a powder had separated. I poured the remaining solution into an evaporating dish and suffered it to evaporate to dryness; but though I alternately applied a gentle and a strong heat for about twenty-four hours, the mass would not become dry, but remained tough, weighing 35 grains.

Exp. XXIII. I endeavoured to dissolve these 35 grains in half an ounce of the strongest alcohol, for which purpose I employed a gentle heat, but on shaking the mixture without having exposed it to any heat, the alcohol was tinged dark red brown, and on giving it the warmth of digestion the greatest part was dissolved; the third part however, though repeatedly treated in a boiling heat with 2 ounces of alcohol, could not entirely be dissolved, but a reddish powder remained, which thrown on living coals spread no considerable smell.

[ To be continued. ]

